# What Should Be the Topics of a Prospective Study on Ovarian Masses in Children?—Results of a Multicenter Retrospective Study and a Scoping Literature Review

**DOI:** 10.3390/curroncol29030125

**Published:** 2022-02-28

**Authors:** Justyna Łuczak, Maciej Bagłaj, Piotr Dryjański, Alicja Kalcowska, Nastazja Banaszyk-Pucała, Maria Boczar, Krzysztof Dymek, Małgorzata Fryczek, Kaja Giżewska-Kacprzak, Wojciech Górecki, Andrzej Grabowski, Anna Gregor, Maria Jabłońska, Grzegorz Kowalewski, Magdalena Lewandowska, Maria Małowiecka, Anna Ogorzałek, Magdalena Pękalska, Aneta Piotrowska-Gall, Mateusz Porębski, Marek Siewiński, Dariusz Patkowski

**Affiliations:** 1 Pediatric Surgery and Urology Department, Wroclaw Medical University, 50-367 Wrocław, Poland; piotrdryjanski@gmail.com (P.D.); a.kalcowska@gmail.com (A.K.); dariusz.patkowski@umw.edu.pl (D.P.); 2 Department of Propedeutic of Pediatrics and Rare Diseases, Wroclaw Medical University, 50-367 Wrocław, Poland; maciej.baglaj@umed.wroc.pl; 3 Pediatric Surgery Department, Dolnośląski Szpital Specjalistyczny im. T. Marciniaka, 54-049 Wrocław, Poland; 4 Department of Pediatric Surgery, Urology and Traumatology, Poznan University of Medical Sciences, 61-701 Poznań, Poland; nastazja90@wp.pl; 5 Clinic of Pediatric Surgery, Institute of Mother and Child, 01-211 Warszawa, Poland; mariaboczar@vp.pl; 6 Department of General and Oncological Pediatric Surgery for Children and Adolescents, Nicolaus Copernicus University, Ludwik Rydygier Collegium Medicum, 85-067 Bydgoszcz, Poland; pikespeak@vp.pl; 7 Pediatric Surgery Department, Jagiellonian University Medical College, 31-008 Kraków, Poland; gosia.grochot@gmail.com (M.F.); wjgorecki@gmail.com (W.G.); 8 Department of Pediatric and Oncological Surgery, Urology and Hand Surgery, Pomeranian Medical University, 70-204 Szczecin, Poland; k.gizewska@gmail.com; 9 Department of Children Developmental Defects and Traumatology, Medical University of Silesia, 40-055 Katowice, Poland; agrabowski@szpital.zabrze.pl; 10 Pediatric Surgery and Urology Department, Zielona Góra Medical University, 65-046 Zielona Góra, Poland; an.gregor@wp.pl; 11 Department of Pediatric Surgery and Organ Transplantation, The Children’s Memorial Health Institute, 04-730 Warszawa, Poland; jablonska.maryla@gmail.com (M.J.); g.kowalewski@ipczd.pl (G.K.); 12 Department of Pediatric Surgery and Oncology, Central University Hospital, Medical University of Lodz, 90-647 Łódź, Poland; magdalena.anna.lewandowska@umed.lodz.pl; 13 Pediatric Surgery Department, Children’s Hospital in Dziekanów Leśny, 05-092 Dziekanów Leśny, Poland; mmmalowiecka@gmail.com; 14 Pediatric Surgery and Urology Clinic, Medical College of Rzeszów University, 65-959 Rzeszów, Poland; ogorzalki@poczta.fm; 15 Chair and Department of Pediatric Surgery and Traumatology, Medical University of Lublin, 20-059 Lublin, Poland; magdalena.pekalska@hotmail.com; 16 Department of Pediatric Surgery, Urology and Traumatology, Collegium Medicum, Jan Kochanowski University, 25-369 Kielce, Poland; md.apiotrowska@gmail.com; 17 Pediatric Surgery and Urology Clinic, Medical University of Silesia, 40-055 Katowice, Poland; mateusz.aleksander.porebski@gmail.com; 18 Pediatric Surgery Department, Opole University Hospital, 45-052 Opole, Poland; marsiew63@gmail.com

**Keywords:** ovarian neoplasms, ovarian masses, ovarian cysts, child, prospective studies, scoping study, therapeutics, evidence-based practice, quality improvement

## Abstract

Purpose: to determine management problems of ovarian masses in girls in order to form a baseline for prospective randomized studies of the established topics and quality improvement of our management. Materials and Methods: We performed a national analysis of clinical aspects of ovarian masses in girls operated on in Poland, analyzed retrospectively medical files of all consecutive patients aged 0–18 who underwent surgeries for ovarian lesions between 2012 and 2017 at 17 pediatric surgical departments and complemented the analysis with a scoping review of a recent primary research related to ovarian masses in children. Results: The study group comprised 595 patients. Forty-four (7.39%) girls were diagnosed with malignant tumors. The overall preservation rate was 64.54%. The analysis revealed that positive tumor markers (OR = 10.3), lesions larger than 6 cm (OR = 4.17) and solid mass on ultrasound examination (OR = 5.34) are interdependent variables differentiating malignant tumors from non-malignant lesions ($X_{4}^{2}$ = 79.1; *p* = 0.00000). Our scoping review revealed 10 major branches of research within the topic of ovarian masses in pediatric population. Conclusions: We have developed an overview of the field with the emphasis on the local environment. Our next step is a multi-institutional prospective study of a quality improvement project implementation based on the obtained knowledge.

## 1. Introduction

An important measure of general health and social well-being is overall reproductive health. For this reason, it is of great importance to preserve fertility in children suffering from ovarian lesions and undergoing treatments affecting ovaries. A pediatric surgeon faced with ovarian mass in a child has to consider this issue while planning a surgery. Although we are aware of the fertility reduction resulting from extensive ovarian surgery, we cannot and we should not forget about malignancy risk. The overarching question inseparably linked to the treatment of ovarian masses in children is how to perform ovarian sparing surgery in every possible case without compromising oncologic principles when needed.

All those who provide health care to children strive to ensure the highest possible quality of care. Moreover, our job as health care professionals is not only to provide care but also to improve it. The management of ovarian lesions varies in demographic, hospital, and physician factors [1]. For many years, there have been no treatment guidelines dedicated to children. A few studies have recently made an attempt to create these; however, no prospective randomized trial exists to confirm their utility [2,3,4]. Our knowledge of ovarian pathologies in children is still far from complete, and much remains to be discovered. To improve care for our patients, it is necessary to find the best evidence and link that evidence of best care with specific knowledge of the local system where that care is provided [5].

Therefore, we have decided to perform a national analysis of clinical aspects of ovarian masses in girls operated on in Poland, complemented with a scoping review of the literature. Based on our 5-year experience, we aimed to determine care problems and to form a baseline for quality improvement of our management as well as a baseline for prospective randomized studies of the established topics.

## 2. Materials and Methods

We retrospectively analyzed medical files of all consecutive patients aged 0–18 who underwent surgical procedures for ovarian lesions between 2012 and 2017 at referral pediatric surgery departments in Poland. This retrospective study was performed using 17 pediatric surgery department databases. We followed the STROBE (strengthening the reporting of observational studies in epidemiology) Checklist. Patients with a paraovarian cyst and those with ovarian torsion without an accompanying lesion were excluded. Demographic data, presenting symptoms and signs, results of laboratory and diagnostic studies (including ultrasound examination, additional imaging studies and tumor markers), and details of surgical procedures and clinical outcomes (including preservation rate), were extracted in each case. Ovarian mass characteristics were evaluated by preoperative imaging (structure and size) or by description of the procedure (size). An ovarian lesion was described arbitrarily as large when its diameter was 10 cm or more in girls aged between 1 and 18 years and 5 cm or more in newborns and infants. Such classification was based on previous experience of other authors in order to obtain comparable results [2,3,4,6,7]. The choice of operative technique (either laparoscopic or open) depended solely on surgeon’s preference. The extent of gonadal resection was based on intraoperative findings and ranged from total, when the whole gonad affected by the lesion was removed, to partial resection, when at least a remnant of ovarian tissue was preserved. Preservation rates were compared, taking into consideration the operative method, the histological type, the size of the mass, the presence of ovarian torsion, and the age of the patient (>1 vs. 1≤). A final diagnosis was made on the basis of a pathology report. All clinical characteristics were reviewed to test their association with malignancy. Therefore, the study group was divided into two subgroups of patients: girls with tumor-like lesions combined with benign tumors (non-malignant group) and malignant tumors. We complemented the described analysis with a scoping review of the recent primary research related to the topic of ovarian masses in children. Based on the results, we identified the topics of the future prospective studies.

### 2.1. Statistical Analysis

Parameters in groups were expressed as median and quartiles or as mean and standard deviation. The statistical significance between different groups was calculated with one-way analysis of variance (ANOVA), alternatively using the non-parametrical U Mann–Whitney* test (for two groups) or Kruskal–Wallis ** test (for more than two groups), when the variances in groups were not homogeneous (the homogeneity of variance was determined with Bartlett’s test). The statistical significance between frequencies was calculated with the chi-square test *χ*^2^_df_ with Yates correction, with corresponding degree of freedom df (df = (m − 1) × (n − 1)), where m—number of rows, and n—number of columns. The statistical significance between frequencies for dependent variables was calculated with McNemara’s test. A multivariate analysis was performed using logistic regression (quasi-Newton model). A *p* value of less than 0.05 was required to reject the null hypothesis. Statistical analysis was performed using EPIINFO Ver. 7.1.1.14 (2 July 2013) software packages.

### 2.2. The Scoping Review

The scoping review followed the methodological framework developed by Arksey and O’Malley and incorporated additional scoping review recommendations made by Levac et al. [8,9]. The protocol is available on request from the corresponding author. We followed the Preferred Reporting Items for Systematic reviews and Meta-Analyses extension for Scoping Reviews (PRISMA-ScR) Checklist (Supplementary File S1). This scoping review relates to the recent primary research (last 10 years). To express the real current state-of-the-art of the topic, we decided to include only this kind of study in our review. We also searched only for studies comprising at least 200 children and regarding the whole spectrum of pathologies. Such an approach was chosen to make the results comparable to our primary study. Our review was conducted in five broad stages, each of which is outlined in the Supplementary File S2. The search flow is also demonstrated in Supplementary File S2.

## 3. Results

### 3.1. Epidemiology and Presentation

The study group comprised 595 patients. The median age was 13.2 ± 5.8 years. Non-malignant masses were noted in 551 girls, including 286 girls (48.07%) with non-neoplastic lesions and 265 girls (44.54%) with benign tumors. Forty-four patients (7.39%) presented with malignant tumors. Table 1 depicts characteristics of each surgical center. There was no significant difference between the non-malignant and malignant group when age at presentation was factored into the analysis (*p* = 0.570). There were no malignant tumors in girls younger than 1 year of age. Mature teratoma was the most frequent lesion among benign neoplasms. The most common malignant lesion noted in our study was juvenile granulosa cell tumor. The histological distribution of all ovarian lesions is presented in Table 2. In accordance with recent studies, pure immature teratomas should be classified as benign tumors [10]. This is a retrospective study, and we cannot verify the results of the pathology report. Taking into consideration that many of the immature teratomas in this study were of stage III at least, it is questionable whether other malignant components were present. Therefore, we included them in the group of malignant lesions.

Palpable mass, abdominal pain, and abdominal distension were the most frequent clinical features noted in the whole study group. Abdominal distension and other symptoms (including: preterm maturation, vaginal bleeding, urinary difficulties, nausea/vomits, and general symptoms such as fever, weight loss, or weakness) were the manifestations that significantly predominated in girls with malignant tumors ($X_{2}^{2}=4.2$; p=0.00084 and X=19.2; p=0.00001 respectively). The main presented symptoms in both groups are shown in Table 3.

### 3.2. Diagnostic Studies

Abdominal ultrasound scans (USs) showed a cystic structure in 315 girls with a non-malignant lesion (58.33%). A heterogeneous ovarian lesion was noted in 196 (36.30%) and a solid mass in further 29 (5.37%) girls from this subgroup. In the malignant group, 22 girls presented with a complex tumor (56.41%). In two of them, ovarian lesions were predominantly cystic. In a further 15 girls (38.46%), a solid structure of the mass was found.

In the whole study group, 250 girls (42.02%) had computer tomography (CT) or magnetic resonance imaging (MRI) studies performed preoperatively. The results of additional examinations (CT/MRI) were not significantly different from the US results (McNemara’s test). Thus, the structure of the mass on US was confirmed with additional imaging in 46 of 67 cystic cases, 96 of 111 complex cases, and 19 of 27 solid cases.

The results of tumor marker evaluation (AFP—alpha-fetaprotein, β-hCG—beta subunit of human chorionic gonadotropin (CA125—cancer antigen 125, LDH—lactate dehydrogenase) were available for 446 girls (74.96%); however, not all of them were tested in each case. They were elevated in 40 girls above 1 year of age (10.72%) with a non-malignant mass. Twenty-five patients with malignant tumor had positive markers (59.52%).

### 3.3. Large Lesions

In total, tumors were defined as large (based on the results of preoperative imaging studies and intraoperative findings) in 219 cases (38.35%). There were statistically significant differences between the groups when size was factored into the analysis. Malignant tumors were larger (p=0.00000).

### 3.4. Bilateral Lesions

Thirty-six girls had bilateral masses (6.05%). There were 8 metachronous lesions (no malignant tumor in this group) and 28 synchronous lesions (six were malignant). Bilateral masses occurred more often in the malignant group ($X_{2}^{2}=4.2$; p=0.0112).

### 3.5. Torsion

Ovarian torsion was noted in 136 patients (22.93%), and its occurrence dominated in the non-malignant group (133 vs. 3 cases). The difference between the groups was statistically significant ($X^{2}=7.17$; p=0.0278).

### 3.6. Treatment

Overall, 276 girls (46.39%) had laparotomy and 270 (45.38%) had laparoscopy performed as an initial operative approach. Conversion to open procedure was noted in 49 girls (8.23%). Thirty-three girls with malignant tumor were subjected to formal laparotomy. The treatment of malignant neoplasms in Poland followed recent oncological guidelines. The management is chosen by an oncological team based on the current treatment protocols and the analysis of recurrence risk factors.

Girls with malignant tumors were excluded from the analysis concerning ovarian tissue preservation. The ovarian tissue-sparing technique (preservation of the ovarian tissue of the affected gonad) was applied in 77.41% patients operated on with the laparoscopic technique and in 45.49% of girls in whom an open procedure was performed. A formal open approach was chosen in 134 patients (68.72%) with large ovarian masses. The preservation rate of ovarian tissue in large lesions was 42.56% compared to 70.37% in the remaining group ($X^{2}=39.2$; p=0.00000). Nevertheless, the preservation rate exceeded 50% in the case of large masses when laparoscopy was used and was around 30% when the open technique was applied.

When age was factored into the analysis, we noted that in girls younger than 1 year of age with a lesion that was not described as having a large preservation rate was at the level of 53.33% with laparoscopy and 33.33% with laparotomy.

Preservation rates were significantly lower when ovarian torsion was present, with the exception of patients older than 1 year of age operated on with laparotomy (Table 4).

### 3.7. Factors Excluding Malignancy

Factors significant in the univariate analysis (tumor markers—negative and positive, age—8 years, size of the lesion—6 cm, US examination result, CT/MR examination result) were subjected to multivariate logistic regression analysis (quasi-Newton model). It revealed that positive tumor markers (OR=10.3), size of the lesion larger than 6 cm (OR=4.17), and solid mass on US examination (OR=5.34) are interdependent variables differentiating malignant tumors from non-malignant lesions ($X_{4}^{2}=79.1$; p=0.00000). A summary of clinical data in the two groups of patients with non-malignant and malignant lesion is presented in Table 3.

### 3.8. Results of the Scoping Study

We identified 16 studies from 8 countries. Fourteen studies were retrospective, and one was prospective. The journal’s title, lead author, place of origin, year of publication, title, study type, population, age group, study group, aims, overview of the methods, outcome measures, and main results related to each study are presented in Supplementary File S3. We also included future study questions indicated by authors and a summary of two systematic reviews that met our criteria concerning the topic. We considered them to be helpful as, to our knowledge, these are the only systematic reviews regarding all kinds of ovarian masses in the last 10 years, and this type of research has a higher level of evidence (Level V) than any separate retrospective study [2,4,6,7,11,12,13,14,15,16,17,18,19,20,21,22,23,24].

Our charting exercise revealed 10 major branches of research within the topic of ovarian masses in pediatric population: epidemiology and presentation, tumor markers, choice of the imaging method, risk factors for malignancy, risk of torsion and its low malignancy risk, use of laparoscopy, surgeon’s specialty and outcome, rate of oophorectomy, and need for prospective study. Additional topics less frequently mentioned or not indicated as the main aim of the studies were as follows: risk factors for oophorectomy, influence of unilateral oophorectomy on fertility, staging, safety of ovarian preservation, second look for unexpected malignancy, multidisciplinary approach for malignancy, length of follow-up, bilateral masses, recurrence/metachronous disease, and management algorithm. A lack of randomized studies in pediatric populations means that almost all the key topics constitute knowledge gaps. An overview of the study topics and a summary of the results are presented in Supplementary File S2.

## 4. Discussion

Most of our local data confirmed the previously obtained knowledge. A high percentage of non-neoplastic lesions among all ovarian masses, a relatively low malignancy risk especially for ovarian torsion, the importance of preoperative imaging with emphasis on solid components or the size of the tumor, and the significance of positive tumor markers results were also revealed by other retrospective studies [2,4,6,7,11,12,13,15,17,18,19,20,21,23,24]. However, some other results raised questions that need to be answered. They were either different from other studies or not discussed by them at all: What is the cut-off size of the lesion indicating malignant tumor?; What is the significance of additional imaging and when is it indicated?; How should the presence of bilateral lesion change our approach?; Why was the preservation rate notably low in children who had laparotomy?; Why was the preservation rate higher for ovarian torsion cases in older children (between 1 and 18 years) when laparotomy was chosen as the operative technique?

Several papers have recently established treatment algorithms for ovarian masses in children [2,3,4]. Their authors considered the size of the lesion an important factor. In a multivariate logistic regression analysis of our national data, we revealed that size of the lesion larger than 6 cm is an interdependent variable differentiating malignant tumors from non-malignant lesions. Whether there is any specific cut-off size for malignancy still needs to be determined. Unfortunately, our study reveals a significant delay in diagnosis of ovarian malignancy in girls despite their obvious presentation. Abdominal distension was noted more often in the case of a malignant mass. This issue indicates some degree of negligence by the patients themselves, their parents, or some medical practitioners. How to raise awareness among adolescent girls, their parents, and healthcare professionals about potential ovarian mass in the case of abdominal enlargement remains a very delicate but important issue that should also be included in the future research.

Another factor questioned by many studies is the significance of tumor markers’ evaluation. The only prospective study in our review excluded them from the management algorithm. Our results revealed their positive value in predicting the histology of ovarian lesions (however, lack of unified testing methods was a relevant limitation in our study). Moreover, they play an important role in the follow-up of malignant lesions. Thus, until more prospective clinical trials regarding this topic are conducted, it seems reasonable to test tumor markers before surgical intervention, preferably in a panel [2,4,21,25].

A US scan is the study of choice during initial assessment of girls with ovarian pathology. The need for further imaging beyond ultrasound in other benign ovarian diseases is not clear. Our study revealed that a CT/MRI result is not an interdependent variable differentiating malignant tumors from non-malignant lesions, and there were no significant differences between the US and additional imaging results. Moreover, additional imaging has some important limitations. Firstly, it may require sedation in a younger child. The availability of pediatric sedation and the radiologist’s confidence in interpreting the imaging examination might influence management and its timing. This might be a critical factor, especially in the case of an ovarian torsion. Secondly, CT scans are a source of radiation, which should be avoided in children in every possible case. On the other hand, US examination depends even more on the radiologist’s experience. The standardization of pelvic ultrasound reports and CT/MRI imaging protocols while conducting future studies might be a key to success in final evaluation of its utility [3,13,17,22,24].

Bilateral masses constitute a serious difficulty in decision making, as do synchronous and metachronous lesions. Performing unnecessary oophorectomy poses a serious risk of infertility if the patient develops a metachronous disease or suffers from ovarian torsion in the future. Another obstacle is the risk of malignancy. In our study, this risk was higher for bilateral lesions. Assessing the real incidence and epidemiology of this specific clinical situation seems to be relevant to help reliably inform and guide the surgeon as well as the patient on the merits of performing safe ovary-sparing surgery. Follow-up guidelines should also be included [4,18,19].

The last two of the above mentioned questions indicate the importance of our attention to the operative techniques. Unfortunately, this is a retrospective study, and there may be factors that contributed to surgical decision making that were not documented. The reported use of tissue sparing procedures varies across the studies (21–96%) [2,6,7,11,12,13,15,17,18,20,23,24]. In our material, the overall preservation rate was 64.54%: a result that leaves much to be desired. However, the use of the laparoscopic technique was associated with a higher preservation of ovarian tissue. This corresponds with other authors recommending this method in the treatment of tumor-like and benign masses [11,26]. What is the possible use and safety of minimally invasive procedures for malignant tumors remains an open question. The bigger size of solid lesions constitutes the most common obstacle for the use of laparoscopy. However, much depends on the surgeons’ experience, and the removal of small first-stage ovarian tumors is suggested to be safe in adult patients where the disease tends to be much more aggressive. The lack of randomized studies seems to be the only obstacle for the use of MIS (minimally invasive surgery) in the treatment of small malignant ovarian lesions. However, one should not forget about its possible usefulness in the staging procedure. Poor adherence to staging guidelines for children with malignant ovarian tumors was reported in the literature, and its correctness is a guarantee of the appropriate postoperative therapy [27,28,29,30]. Last but not least, the results obtained by our study revealed a disturbingly low preservation rate in children younger than 1 year of age, especially for laparotomy cases. In addition, there was no malignant tumor in this age group, reflecting a probably lower malignancy risk in these girls. Treatment of ovarian cysts in children under 12 months has also recently become less invasive, with the aim of sparing ovarian tissue by follow-up without intervention, or with the use of ovary-preserving surgeries even in patients with torsion. Such an approach should be introduced immediately and widely [31].

Only two studies from our scoping review addressed the issue of follow-up of patients. It is of no surprise that evaluating late outcomes in pediatric population poses difficulties. With a view to potential serious negative effects of the surgical treatment on fertility, a lack of interest in the influence of oophorectomy on the fertility of patients among the studies is disturbing. Only one of the reviewed papers confronted this topic [12,19].

The aforementioned questionable issues obviously require further research. Although obtaining sound data from well-conducted studies is of crucial value, an important component of our everyday clinical practice is the collaboration between experts in the field. Recent studies have reported differences in the management strategies across specialties. In the face of this knowledge, we must not forget about the value of the multidisciplinary approach. In particularly doubtful cases, seeking advice might help in implementing a successful sparing surgery, identifying a malignant lesion, choosing the best postoperative treatment, or planning an appropriate follow-up of the patient [3,12].

Our study poses several limitations. The retrospective design forces a reliance on documentation of patient symptoms by the charting physician. In addition, the particular intraoperative decisions of the surgeons were not documented. The use of a scoping review methodology was particularly advantageous, as we could choose some specific inclusion criteria. However, we were limited in a number of ways. We were probably unable to find all relevant studies. A quantitative synthesis may have revealed additional insights. Not encompassing all studies concerning ovarian masses in more specified study groups (e.g., only malignant tumors included, only specific neoplasms included) hindered our ability to fully analyze the topic in the context of more thematic restricted studies.

The research evidence on ovarian lesions in children is limited. Unfortunately, a paucity of prospective studies dedicated exclusively to children, including all types of ovarian lesions, and comprising a large cohort of patients makes an objective insight into these aspects difficult. Our scoping review revealed an insufficient number of basic research and prospectively designed high-quality multi-institutional studies. Strong experimental design and accurate data analysis are vitally important to build new generalizable scientific knowledge. However, evidence-based practice is not synonymous with research evidence. It is a process of shared decision making between a practitioner, a patient, and others important for them based on research, as well as the complete picture of the needs of an individual patient, clinical expertise, and other available sources of information [5]. We believe that introducing a unified treatment guideline in our practice is obligatory and necessary. We aim to improve the quality of our management strategy, primarily with a view to increase preservation rates while maintaining oncologic principles. Improvement interventions have become an increasingly important focus of activity within healthcare. They can be defined broadly as purposeful efforts to secure positive change. There have been studies highlighting unsatisfactory outcomes of the treatment of pediatric masses in children where implementation of quality improvement projects led to positive effects [3,32,33,34].

## 5. Conclusions

This primary study complemented with a scoping review of the literature allowed us to develop an overview of the field, with the emphasis on our local environment. We distinguished the following main branches of research within the topic of ovarian masses in pediatric population: epidemiology and presentation (including the incidence and age distribution), tumor markers (including their types and role in excluding malignancy), choice of the imaging method (including the characteristic features of malignant masses on US examination and the significance and indications for the use of CT and MRI), risk factors for malignancy, risk of torsion and its low malignancy risk, use of laparoscopy (including its role in managing malignant tumors and large masses), surgeon’s specialty and outcome, rate of oophorectomy (including factors that influence the preservation of ovarian tissue), and management in cases of bilateral lesions. We also enumerated several additional topics. The presented study will serve as the basis for future research. Our next step is a multi-institutional prospective study of the implementation of a quality improvement project including an ovarian mass management algorithm suitable for our local system, standardized documentation and follow-up of patients, evaluation of the rest of the above distinguished topics, training initiatives, and any other possible factor revealed through the multidisciplinary discussion of the local experts in the field. This future research is needed to identify the potential harms of the treatment methods and to evaluate their effectiveness, as well as to find answers to the knowledge gaps identified in this study and during the preparation of the project.

## Figures and Tables

**Table 1:** Characteristics of each surgical center.

City	Number of Patients	Histological Distribution:			Preservation Rate
		Tumor-Like	Benign Neoplasm	Malignant Neoplasm	
Warsaw 1	105	57	42	6	59.4%
Lodz	26	15	10	1	61.5%
Warsaw 2	20	20	0	0	10.0%
Zabrze	40	27	12	1	70.0%
Katowice	30	4	24	2	40.0%
Kielce	30	8	17	5	69.2%
Szczecin	22	16	5	1	68.2%
Poznan	43	24	14	5	69.5%
Wroclaw 1	62	35	22	5	48.4%
Krakow	65	8	51	6	87.7%
Wroclaw 2	17	10	6	1	45.5%
Olsztyn	28	16	11	1	92.8%
Opole	16	4	9	3	20.0%
Rzeszow	44	20	21	3	63.2%
Zielona Gora	5	4	1	0	75.0%
Bydgoszcz	14	1	10	3	28.6%
Lublin	28	17	10	1	50.0%

Warsaw 1 (Department of Pediatric Surgery and Organ Transplantation, The Children’s Memorial Health Institute). Warsaw 2 (Clinic of Pediatric Surgery, Institute of Mother and Child). Wroclaw 1 (Pediatric Surgery and Urology Department, Wroclaw Medical University). Wroclaw 2 (Pediatric Surgery Department at Dolnośląski Szpital Specjalistyczny im. T. Marciniaka).

**Table 2:** Histological distribution.

	Type of the Lesion (No. of Patients)	Pathology Report (No. of Patients)	Median Age (Lower ÷ Upper Quartile)
Non-malignant lesions	TUMOR-LIKE LESIONS (**286**)	Simple cyst (**188**)	**13.2 (8.0 ÷ 15.5) years**
		Hemorrhagic cyst (**89**)	
		Endometriosis (**9**)	
	BENIGN TUMORS (**265**)	Mature teratoma (**189**)	
		Epithelial tumor * (**72**)	
		Fibroma (**4**)	
Malignant lesions	GERMINAL TUMORS (**22**)	Dysgerminoma (**6**)	**13.1 (10.5 ÷ 15.0) years**
		Immature teratoma (**5**)	
		Endodermal sinus tumor (**4**)	
		Mixed germ cell tumor ** (**7**)	
	STROMAL TUMORS (**12**)	Juvenile granulosa cell tumor (**10**)	
		Sertoli cell tumor (**1**)	
		Serous papillary carcinoma (**1**)	
	EPITHELIAL TUMORS (**4**)	Papillary mucinous cysadenocaricnoma (**1**)	
		Adenocarcinoma (**1**)	
		Small cel carcinoma (**1**)	
		Gynandroblastoma (**1**)	
	OTHER (**6**)	Choriocarcinoma (**1**)	
		Burkitt’slymphoma (**2**)	
		PNET (**1**)	
		Desmoplastic Small Round Cell Tumor (**1**)	

* cystadenofibroma, serous cystadenoma, mucinous cystadenoma; ** including yolk sac.

**Table 3:** Summary of the clinical data.

		Malignant	Non-Malignant	*p* Value (When Statistically Significant)	Multivariate Analysis
Number of Patients		44	551		
**Age (years)**	median	13.1 (10.5 ÷ 15.0)	13.2 (8.0 ÷ 15.5)		
	0–1	0	93/16.88%		
	2–4	2/4.55%	23/4.17%		
	5–8	5/11.36%	35/6.35%		
	9–14	23/52.27%	212/38.48%		
	15–18	14/31.82%	188/34.12%		
	<8 years	5/11.36%	135/24.50%	χ^2^ = 3.91 *p* = 0.0480	
	≥8 years	39/88.64%	416/75.50%		
**Symptoms**	abdominal pain	21/47.73%	325/59.98%		
	palpable mass	14/31.82%	134/24.32%		
	distension	15/34.09%	76/13.79%	χ^2^_2_ = 14.2 *p* = 0.00084	
	other	9/20.45%	25/4.54%	χ^2^ = 19.2 *p* = 0.00001	
**Us result**	solid	15/38.46%	29/5.37%	χ^2^_2_ = 75.1 *p* = 0.00000	OR = 5.34
	complex	22/56.41%	196/36.30%		
	cystic	2/5.13%	315/58.33%		
**Ct/mri result**	solid	15/46.875%	10/5.235%	χ^2^_4_ = 122.8 *p* = 0.00000	
	complex	16/50.00%	112/58.64%		
	cystic	1/3.125%	69/36.125%		
**Size of the lesion**	median	12.5 (8.7 ÷ 16.5)	6.00 (5.0 ÷ 9.0)	*p* = 0.00000	
	large lesion	29/69.05%	160/30.77%		
	lesion that was not described as large	13/30.95%	360/69.23%		
	<6 cm	5/11.90%	204/38.56%	χ^2^ = 11.9 *p* = 0.00056	
	≥6 cm	37/88.10%	325/61.44%		OR = 4.17
**Tumor markers**	positive	24/41 (58.54%)	40/405 (9.88%)	χ^2^ = 71.7 *p* = 0.0000	OR = 10.3
	negative	17/41 (41.46%)	365/405 (90.12%)		
**Bilateral lesion**		6/13.64%	30/5.44%	χ^2^_2_ = 8.98 *p* = 0.0112	
**Ovarian torsion**		3/6.82%	133/24.14%	χ^2^ = 7.17 *p* = 0.0278	
					χ^2^_4_ = 79.1 *p* = 0.00000

**Table 4:** Preservation rates.

		Laparatomy		Laparoscopy	
		≤1 year	>1 year	≤ 1 year	>1 year
**General**		31.11%	48.15%	62.16%	80.20%
	*p* value	*χ*^2^ = 7.91; ***p* = 0.00493**	*χ*^2^ = 48.4; ***p* = 0.00000**	*χ*^2^ = 7.91; ***p* = 0.00493**	*χ*^2^ = 48.4; ***p* = 0.00000**
**Size of the mass**	small	33.33%	61.76%	53.33%	81.99%
	large	31.43%	29.00%	69.57%	73.68%
	*p* value	*χ*^2^_3 _= 29.00; ***p* = 0.00000**		*χ*^2^_3 _= 8.06; ***p* = 0.0447**	
**Torsion**	present	10.00%	46.67%	30.77%	63.89%
	absent	48.00%	48.63%	79.17%	83.73%
	*p* value	*χ*^2^ = 7.4; ***p* = 0.00622**	*χ*^2^ = 0.0700; *p* = 0.791	*χ*^2^ = 8.4; ***p* = 0.00376**	*χ*^2^ = 7.34; ***p* = 0.00675**
		**OVERALL**			
		≤1 year	>1 year		
**Size of the mass**	small	70.37%			
	large	42.56%			
	*p* value	*χ*^2 ^= 39.2; ***p* = 0.00000**			
**Torsion**	present	18.18%	53.15%		
	absent	63.27%	65.33%		
	*p* value	*χ*^2^ = 16.2; ***p* = 0.00006**	*χ*^2^ = 4.79; ***p* = 0.0285**		

## Data Availability

The datasets used and analyzed during the current study are available from the corresponding author on reasonable request.

## Supplementary Materials

The following supporting information can be downloaded at: https://www.mdpi.com/article/10.3390/curroncol29030125/s1, Supplementary File S1—PRISMA-ScR Checklist; Supplementary File S2—Methodology and results of the scoping study; Supplementary File S3—Relevant data from each source of evidence.
