# Supplementary material for: What Should Be the Topics of a Prospective Study on Ovarian Masses in Children?—Results of a Multicenter Retrospective Study and a Scoping Literature Review
Source: Curr Oncol. 2022 Feb 28;29(3):1488–500. doi: 10.3390/curroncol29030125 (PMC8946882; doi:10.3390/curroncol29030125)
Supplement: Supplementary file 1 [file curroncol-29-00125-s001.zip › Supplementary File S2 - Methodology and results of the scoping study.pdf]

Methodology and Results of the Scoping Study

1. Stages of the Scoping Study

1.1. Stage 1: identifying the Research Questions

As we are becoming increasingly familiar with the literature, we have identified the following guiding questions in the area:

- What are the key topics covered by the selected studies?
- What are the topics that are most amenable?
- Are there certain areas of ovarian masses in children that can be explored more thoroughly than others?
- What are the key gaps in the existing knowledge?
- Which areas need more research?
- Are there reasons for certain areas being under-researched?

1.2. Stage 2 and 3: Identifying and Selecting Relevant Studies

The database search was run on 10 September by one of the authors. Article selection and review took approximately one month and was completed by all authors. Final terms were determined after an initial broad search using MEDLINE, which was used to identify MESH headings and alternative terms used in relevant papers. Using formerly described guidelines, in consultation with a subject specialist librarian, we have developed a PubMed-specific search strategy. The following electronic databases have been searched: (1) PubMed; (2) Web of Science (3) CINAHL (4) Cochrane Central Register of Controlled Trials. We also conducted a thorough scan of relevant grey literature (OpenGrey and Google). We limited our search to those with English language abstracts published between 2011 and 2021.

The inclusion criteria and search strategy are shown below in this file. The review team started the process by reviewing together a small sample of studies in order to ensure that there was an agreed common understanding about the inclusion and exclusion criteria. Disagreements about the papers were discussed midway, and at the end of the process. The selection process and search flow is demonstrated below in this file.

1.3. Stage 4: Charting the Data

The data reported in the eligible papers were charted in an Excel spreadsheet. Characteristics included publication details, authors, year of publication, study location, study type (e.g. retrospective study), study group, aims of the study, overview of the methods, outcomes measures and results.

1.4. Stage 5: Collating, Summarizing and Reporting the Results

An overview of all material. We let the content of the included studies guide our theme development and identified and highlighted certain patterns across the papers in our study in the charting exercise. Three distinct steps were conducted: (1) Analysis - including descriptive numerical summary analysis

and thematic analysis; (2) Reporting the results and producing the outcome that refers to the overall purpose or research question; (3) Discussing implications for future research and practice.

We grouped the studies by the type of settings along with the measures used and broad findings. The remainder of this review will present the main points of research within the research questions described above. The review will conclude outlining the knowledge gaps that exist in addressing the primary question.

2. Inclusion Criteria and Search Strategy

Inclusion criteria	• written in English<br>• reports primary research<br>• concerns ovarian teratoma in pediatric age (0-18 years)<br>• does not concern any specific ovarian lesion (e.g. only germ cell tumors)<br>• study date 2011-2021
Keywords considered	Ovarian teratoma: Ovarian Neoplasms; Ovary Neoplasms; Neoplasms, Ovary; Ovarian Cysts; Ovary Cysts; Cysts, Ovary<br><br>Mass: Mass; Lesion; Tumor; Cyst<br><br>Ovary: Ovaries; Gonads<br><br>Child: Adolescent; Child, Preschool; Infant; Children; Minors; Pediatric
Search strategy in PubMed	**1.** (("Ovarian Neoplasms"[Mesh] OR "Cysts"[Mesh]) AND "Child"[Mesh]) OR ((“ovarian masses in children”) NOT medline[sb])<br><br>**2.** (("Ovarian Neoplasms"[Mesh]) OR "Cysts"[Mesh]) AND "Child"[Mesh] OR (ovarian neoplasms* AND cysts* AND child* NOT medline[sb])
Search strategy in Web of Science	1. # 1 (((ALL=(ovarian neoplasms AND ALL=(child)) AND LA:(English)) AND PY=(2011-2021)<br>2. # 2 (((ALL=(ovarian cysts) AND ALL=(child)) AND LA=(English)) AND PY=(2011-2021)<br>3. #3 (((ALL=(ovarian mass)) AND ALL=(child)) AND LA=(English)) AND PY=(2011-2021)<br>4. #4 (((ALL=(ovarian lesion)) AND ALL=(child)) AND LA=(English)) AND PY=(2011-2021)<br>5. #5 (((ALL=(ovarian tumor)) AND ALL=(child)) AND LA=(English)) AND PY=(2011-2021)<br>6. #1 OR #2 OR #3 OR #4 OR #5 AND LA:(English)) AND PY=(2011-2021)

3. Selection Process and Search Flow

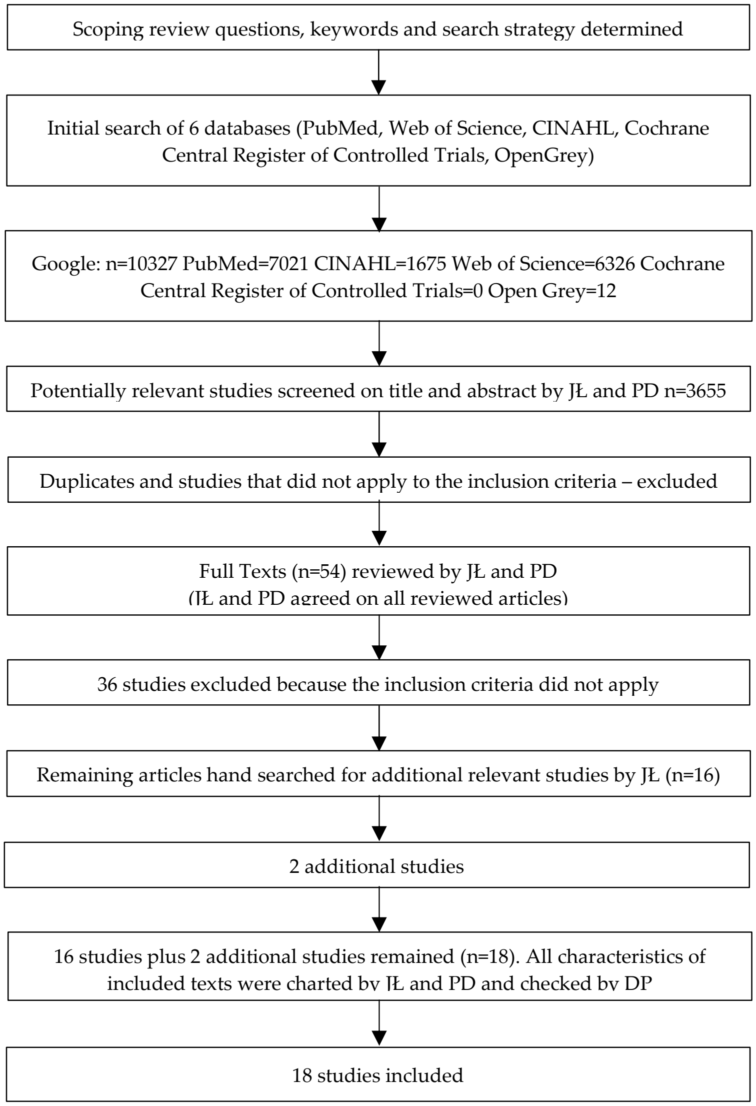

4. Results

4.1. Overview of the Study Topics

TOPIC
(main with red color)

**Article No.**	Epidemiology and presentation	Tumor markers	Choice of the imaging method	Risk factors for malignancy	Risk factors for oophorectomy	Influence of unilateral oophorectomy on	Risk of torsion and its low malignancy risk	Use of laparoscopy	Surgeon’ s speciality and outcome	Staging	Safety of ovarian preservation	Second check for unexpected malignancy	Multidisciplinary approach for	Rate of oophorectomy	Length of follow up	Bilateral masses	Recurrence/Metachronous disease	Management algorithm	Need of prospective study	**Overall topics included**
**1**	1	1	1	1			1	1						1		1	1			**9**
**2**	1							1					1	1	1	1				**6**
**3**	1		1	1			1							1		1				**6**
**4**	1	1		1	1				1			1		1		1			1	**9**
**5**										1										**1**
**6**	1													1						**2**
**7**										1						1			1	**3**
**8**	1		1	1			1	1						1		1		1		**8**
**9**	1	1	1	1			1	1						1		1		1	1	**10**
**10**	1					1								1	1	1	1			**6**
**11**	1	1		1	1				1					1					1	**7**
**12**	1	1	1	1			1	1	1					1		1			1	**10**
**13**	1	1	1	1			1			1	1						1	1		**9**
**14**	1				1			1	1					1		1	1			**7**
**15**	1	1		1															1	**4**
**16**			1																1	**2**
**17**	1	1	1	1	1		1	1	1					1		1				**10**
**18**	1	1	1	1										1		1				**6**
**Overall number of the studies**	**15**	**9**	**9**	**11**	4	1	**7**	**7**	**5**	3	1	1	1	**13**	2	**12**	4	3	**7**	

4.2. Summary of the Results

The above table shows the key topics covered by selected studies. Answering the next two of the study questions: what are the topics that are most amenable and are there certain areas of ovarian masses that can be explored more thoroughly than others? – it seems to be much easier and common to examine the diagnosis and treatment currently applied in the management of ovarian lesions than defining its nature and behavior by means of prospective and experimental studies. Most of the studies left their study questions without clear answers thus indicating the need for further research. Therefore, we answered indirectly one of the other study questions: are there reasons for certain areas to be under-researched? Furthermore, almost all of the studies highlighted their limitations. The majority was of retrospective design thus uniform diagnostic, treatment and follow-up methods across the studies were lacking. Referring to old studies is another important obstacle. Difficulties in studying ovarian masses in children seem to be present in all aspects beginning from their rarity. Evaluating outcomes in pediatric population is another important limitation keeping in mind that many effects of our treatment might be observed only in the adulthood. Only two of the reviewed papers concerned the issue of follow-up. Lack of randomized studies in pediatric population makes actually almost all of the key topics the knowledge gaps. Future research is needed to establish the final management strategy, identify the potential harms of the treatment methods and to evaluate their effectiveness as well as to understand true clinical nature of ovarian lesions in pediatric population [1–18].

5. References

1. Liu, H.; Wang, X.; Lu, D.; Liu, Z.; Shi, G. Ovarian Masses in Children and Adolescents in China: Analysis of 203 Cases. *Journal of Ovarian Research* **2013**, *6* (1), 47. https://doi.org/10.1186/1757-2215-6-47.
2. Cribb, B.; Vishwanath, N.; Upadhyay, V. THE NEW ZEALAND MEDICAL JOURNAL Paediatric Ovarian Lesions-the Experience at Starship Children's Hospital, New Zealand. *Journal of the New Zealand Medical Association NZMJ* **2014**, *6*, 8716.
3. Zhang, M.; Jiang, W.; Li, G.; Xu, C. Ovarian Masses in Children and Adolescents - an Analysis of 521 Clinical Cases. *Journal of pediatric and adolescent gynecology* **2014**, *27* (3). https://doi.org/10.1016/J.JPAG.2013.07.007.
4. Madenci, A. L.; Levine, B. S.; Laufer, M. R.; Boyd, T. K.; Voss, S. D.; Zurakowski, D.; Frazier, A. L.; Weldon, C. B. Preoperative Risk Stratification of Children with Ovarian Tumors. *Journal of pediatric surgery* **2016**, *51* (9), 1507–1512. https://doi.org/10.1016/J.JPEDSURG.2016.05.004.
5. Hermans, A. J.; Kluivers, K. B.; Siebers, A. G.; Wijnen, M. H. W. A.; Bulten, J.; Massuger, L. F. A. G.; Coppus, S. F. P. J. The Value of Fine Needle Aspiration Cytology Diagnosis in Ovarian Masses in Children and Adolescents. *Human reproduction (Oxford, England)* **2016**, *31* (6), 1236–1240. https://doi.org/10.1093/HUMREP/DEW072.
6. Hermans, A. J.; Kluivers, K. B.; Janssen, L. M.; Siebers, A. G.; Wijnen, M. H. W. A.; Bulten, J.; Massuger, L. F. A. G.; Coppus, S. F. P. J. Adnexal Masses in Children, Adolescents and Women of Reproductive Age in the Netherlands: A Nationwide Population-Based Cohort Study. *Gynecologic oncology* **2016**, *143* (1), 93–97. https://doi.org/10.1016/J.YGYNO.2016.07.096.
7. Vadva, Z.; Laufer, M. R.; Weldon, C. B.; Frazier, A. L.; Vargas, S. O. Diagnostic Impact of Peritoneal Fluid Cytology in the Setting of Pediatric Uterine Adnexal Biopsy or Resection. *Pediatric and developmental pathology : the official journal of the Society for Pediatric Pathology and the Paediatric Pathology Society* **2016**, *19* (5), 401–408. https://doi.org/10.2350/15-10-1716-OA.1.

8. Stanković, Z. B.; Sedlecky, K.; Savić, D.; Lukač, B. J.; Mažibrada, I.; Perovic, S. Ovarian Preservation from Tumors and Torsions in Girls: Prospective Diagnostic Study. *Journal of pediatric and adolescent gynecology* **2017**, *30* (3), 405–412. https://doi.org/10.1016/J.JPAG.2017.01.008.
9. Łuczak, J.; Bagłaj, M. Selecting Treatment Method for Ovarian Masses in Children – 24 Years of Experience. *Journal of Ovarian Research* **2017**, *10* (1). https://doi.org/10.1186/S13048-017-0353-0.
10. Braungart, S.; Craigie, R. J.; Farrelly, P.; Losty, P. D. Operative Management of Pediatric Ovarian Tumors and the Challenge of Fertility-Preservation: Results from the UK CCLG Surgeons Cancer Group Nationwide Study. *Journal of pediatric surgery* **2020**, *55* (11), 2425–2429. https://doi.org/10.1016/J.JPEDSURG.2020.02.057.
11. Braungart, S.; Craigie, R. J.; Farrelly, P.; Losty, P. D. Ovarian Tumors in Children: How Common Are Lesion Recurrence and Metachronous Disease? A UK CCLG Surgeons Cancer Group Nationwide Study. *Journal of pediatric surgery* **2020**, *55* (10), 2026–2029. https://doi.org/10.1016/J.JPEDSURG.2019.10.059.
12. Lawrence, A. E.; Gonzalez, D. O.; Fallat, M. E.; Aldrink, J. H.; Hewitt, G. D.; Hertweck, S. P.; Onwuka, A.; Bence, C.; Burns, R. C.; Dillon, P. A.; Ehrlich, P. F.; Fraser, J. D.; Grabowski, J. E.; Hirschl, R. B.; Kabre, R.; Kohler, J. E.; Lal, D. R.; Landman, M. P.; Leys, C. M.; Mak, G. Z.; Sato, T. T.; Scannell, M.; Sujka, J. A.; Minneci, P. C.; Deans, K. J. Factors Associated With Management of Pediatric Ovarian Neoplasms. *Pediatrics* **2019**, *144* (1). https://doi.org/10.1542/PEDS.2018-2537.
13. How, J. A.; Marino, J. L.; Grover, S. R.; Heloury, Y.; Sullivan, M.; Mellor, A.; McNally, O.; Jayasinghe, Y. Surgically Managed Ovarian Masses at the Royal Children's Hospital, Melbourne -19 Year Experience. *Journal of pediatric surgery* **2019**, *54* (9), 1913–1920. https://doi.org/10.1016/J.JPEDSURG.2019.05.005.
14. Renaud, E. J.; Sømme, S.; Islam, S.; Cameron, D. B.; Gates, R. L.; Williams, R. F.; Jancelewicz, T.; Oyetunji, T. A.; Grabowski, J.; Diefenbach, K. A.; Baird, R.; Arnold, M. A.; Lal, D. R.; Shelton, J.; Guner, Y. S.; Gosain, A.; Kawaguchi, A. L.; Ricca, R. L.; Goldin, A. B.; Dasgupta, R. Ovarian Masses in the Child and Adolescent: An American Pediatric Surgical Association Outcomes and Evidence-Based Practice Committee Systematic Review. *Journal of pediatric surgery* **2019**, *54* (3), 369–377. https://doi.org/10.1016/J.JPEDSURG.2018.08.058.
15. Lawrence, A. E.; Fallat, M. E.; Hewitt, G.; Hertweck, P.; Onwuka, A.; Afrazi, A.; Bence, C.; Burns, R. C.; Corkum, K. S.; Dillon, P. A.; Ehrlich, P. F.; Fraser, J. D.; Gonzalez, D. O.; Grabowski, J. E.; Kabre, R.; Lal, D. R.; Landman, M. P.; Leys, C. M.; Mak, G. Z.; Overman, R. E.; Rademacher, B. L.; Raiji, M. T.; Sato, T. T.; Scannell, M.; Sujka, J. A.; Wright, T.; Minneci, P. C.; Deans, K. J.; Aldrink, J. H. Understanding the Value of Tumor Markers in Pediatric Ovarian Neoplasms. *Journal of pediatric surgery* **2020**, *55* (1), 122–125. https://doi.org/10.1016/J.JPEDSURG.2019.09.062.
16. van Nimwegen, L. W. E.; Mavinkurve-Groothuis, A. M. C.; de Krijger, R. R.; Hulsker, C. C. C.; Goverde, A. J.; Zsiros, J.; Littooij, A. S. MR Imaging in Discriminating between Benign and Malignant Paediatric Ovarian Masses: A Systematic Review. *European radiology* **2020**, *30* (2), 1166–1181. https://doi.org/10.1007/S00330-019-06420-4.
17. Wang, Q.; Yu, D.; Wang, F. Clinical and Computed Tomographic Features of Ovarian Lesions in Infants, Children, and Adolescents: A Series of 222 Cases. *Journal of pediatric and adolescent gynecology* **2021**, *34* (3), 387–393. https://doi.org/10.1016/J.JPAG.2020.10.007.
18. Xac, M. C.; Jetelina, K. K.; Jarin, J.; Wilson, E. Benign, Borderline, and Malignant Pediatric Adnexal Masses: A 10-Year Review. *Journal of pediatric and adolescent gynecology* **2021**, *34* (4), 454–461. https://doi.org/10.1016/J.JPAG.2021.01.002.
