# Supplementary figures and images for: What Should Be the Topics of a Prospective Study on Ovarian Masses in Children?—Results of a Multicenter Retrospective Study and a Scoping Literature Review
Source: Curr Oncol. 2022 Feb 28;29(3):1488–500. doi: 10.3390/curroncol29030125 (PMC8946882; doi:10.3390/curroncol29030125)

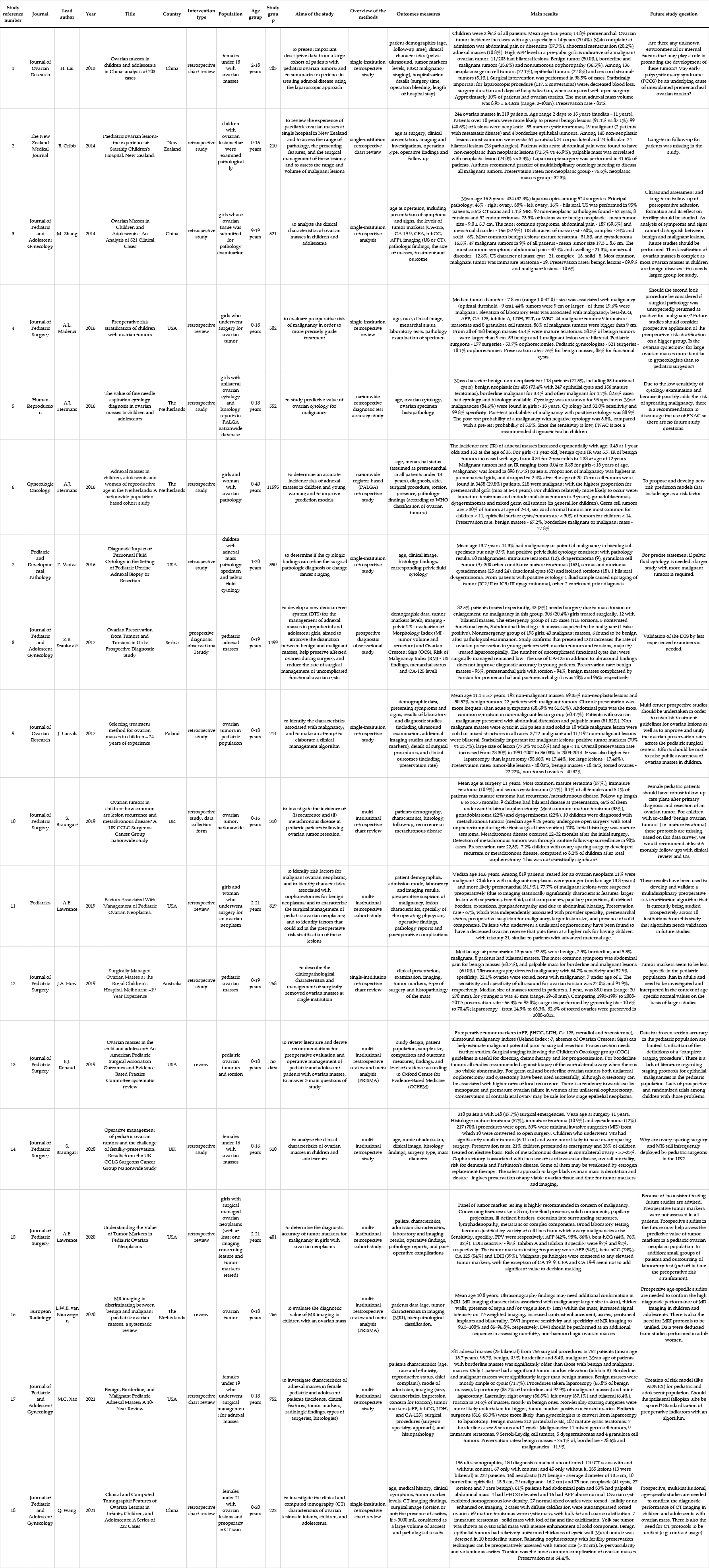

Supplement: Supplementary file 1 [file curroncol-29-00125-s001.zip › Supplementary File S3 - Relevant data from each source of evidence.tif]
